# Traps as treats: a traditional sticky rice snack persisting in rapidly changing Asian kitchens

**DOI:** 10.1186/s13002-015-0010-x

**Published:** 2015-03-24

**Authors:** Rachel Schwallier, Hugo J de Boer, Natasja Visser, Rogier R van Vugt, Barbara Gravendeel

**Affiliations:** Naturalis Biodiversity Center, Darwinweg 2, 2333 CR Leiden, The Netherlands; Uppsala University, Norbyvägen 18D, SE 75236 Uppsala, Sweden; The Natural History Museum, University of Oslo, P.O. Box 1172, NO-0318 Oslo, Norway; University of Applied Sciences Leiden, Zernikedreef 11, 2300 AJ Leiden, The Netherlands; Hortus botanicus of Leiden University, Rapenburg 73, 2311 GJ Leiden, The Netherlands; Institute Biology Leiden, Plant Ecology x Phytochemistry Group, Leiden University, Sylviusweg 72, 2333 CC Leiden, The Netherlands

**Keywords:** Borneo, Carnivorous plants, Ethnobotany, Glutinous rice, *Nepenthes*, Malaysian tribes, Traditional food

## Abstract

**Background:**

An accessory to modern developing economies includes a shift from traditional, laborious lifestyles and cuisine to more sedentary careers, recreation and convenience-based foodstuffs. Similar changes in the developed western world have led to harmful health consequences. Minimization of this effect in current transitional cultures could be met by placing value on the maintenance of heritage-rich food. Vitally important to this is the preservation and dissemination of knowledge of these traditional foods. Here, we investigate the history and functionality of a traditional rice snack cooked in *Nepenthes* pitchers, one of the most iconic and recognizable plants in the rapidly growing economic environment of Southeast Asia.

**Methods:**

Social media was combined with traditional ethnobotanical surveys to conduct investigations throughout Malaysian Borneo. Interviews were conducted with 25 market customers, vendors and participants from various ethnical groups with an in-depth knowledge of glutinous rice cooked in pitcher plants. The acidity of pitcher fluid was measured during experimental cooking to analyze possible chemical avenues that might contribute to rice stickiness.

**Results:**

Participants identifying the snack were almost all (96%) from indigenous Bidayuh or Kadazandusun tribal decent. They prepare glutinous rice inside pitcher traps for tradition, vessel functionality and because they thought it added fragrance and taste to the rice. The pH and chemical activity of traps analyzed suggest there is no corresponding effect on rice consistency. Harvest of pitchers does not appear to decrease the number of plants in local populations.

**Conclusions:**

The tradition of cooking glutinous rice snacks in pitcher plants, or *peruik kera* in Malay, likely carries from a time when cooking vessels were more limited, and persists only faintly in tribal culture today because of value placed on maintaining cultural heritage. Social media proved a valuable tool in our research for locating research areas and in interviewing respondents, and we endorse its further use in ethnobotanical investigations. Our gathered data urges for the preservation of sustainable, tribal plant use for the prosperity of both health and culture.

## Introduction

The culture and preparation of food triggers memories, shapes our everyday life and reflects the priorities and progression of cultures. The developed western world’s current revival of eating local, ‘slow’ food and its trending diet named after our Paleolithic ancestors is an imperative reaction to the rampant crises of obesity, food allergies, cardiovascular disease, type II diabetes and other habit-induced health incursions [1]. These are owned from a long-endured transition in habits acquired during rapid economic growth [2]. Present day countries with freshly advancing economic environments undergo a similar shift from traditional foodstuffs to convenience foods and from laborious jobs and lifestyles to more sedentary careers and recreation [3]. The implications of these changes are apparent at very early stages in these shifting economies [2].

Maintenance of tradition in food preparation is one solution to minimize the gap experienced in the western world. Vitally important to the continued benefits traditional foodstuffs offer, is simply preserving the knowledge involved in its preparation and history [4]. Here, we offer a contribution to this knowledge bank by uncovering the history and culture of a luring glutinous rice snack cooked inside a carnivorous plant trap.

*Nepenthes* L. is an iconic genus from Southeast Asia with modified leaf traps shaped as pitchers that capture and digest their prey. Throughout its growing regions, the pitchers have a functional role in traditional culture [5-8]. *Nepenthes* are medicinally used to relieve gastrointestinal discomfort, including dysentery, stomachache and bed-wetting [9]. They are also used to prevent malaria, and the roots contain plumbagin [10], which shows promising *in vitro* and *in vivo* antiplasmodial efficacy [11]. The plant provides material in housing construction and serves as a protective male sheath in West Papua [12]. Despite their intrigue, the culture of *Nepenthes* in traditional food is not extensively known. Previous accounts of the use of *Nepenthes* in traditional food are framed within larger works of carnivorous plants, food packaging or ethnobotany, which allowed only brief identification of *Nepenthes* in food culture [13-15].

With this study we analyze and present the preparation, culture and significance of the sticky rice snack made inside *Nepenthes* pitchers. Our research was based on the following questions: How and when is the *Nepenthes* sticky rice snack prepared? What is the main motivation for using *Nepenthes* in food preparation? Is there a contribution from the pitcher to chemically induce rice stickiness in the snack? Which species of *Nepenthes* were used in history and now? How does their distribution reflect their usage?

## Materials and methods

### Area of investigation

Documented areas of cooking rice inside *Nepenthes* pitchers included Thailand [14], the Philippines [15] and Malaysia [13]. These accounts gave us a general base, but social media allowed a more detailed current glimpse of the present day culture and whereabouts of this snack. We scoured personal travel blogs, the photo sharing website ^©^Flickr and the visual board tool ^©^Pinterest to uncover that production and consumption of this traditional snack was alive and thriving, especially in the southern parts of Sarawak, Malaysia. We therefore targeted our efforts in Malaysian Borneo, where many indigenous tribes are known to continue their rich traditions [16]. Malaysian Borneo sits in the north of this third largest island in the world. It has one of the oldest and richest rainforests and is considered the main center of biodiversity for *Nepenthes* [17]. Malaysia is a rapidly developing country and, like many ‘aspiring’ economies, is experiencing a shift towards a more sedentary lifestyle and increased consumption of high caloric processed foods [2].

### Data collection

**Figure 1:**
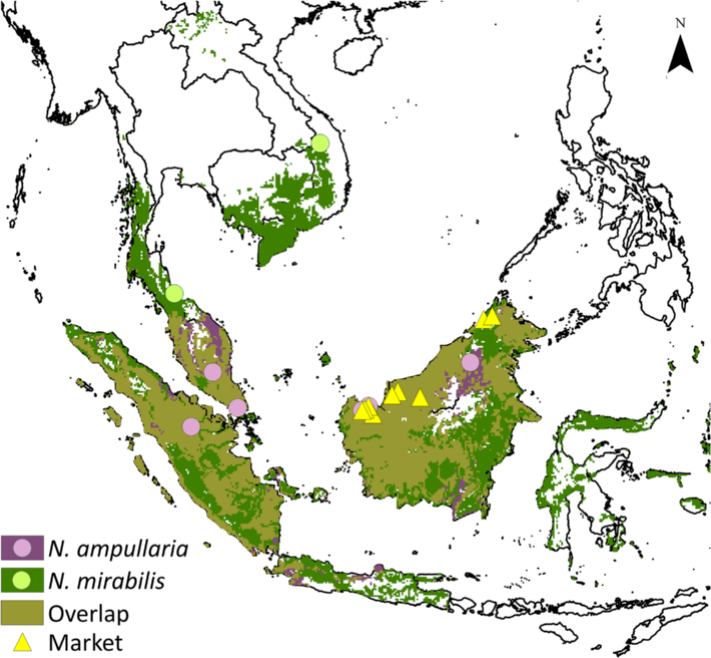
**Glutinous rice snack localities and predicted distribution of pitcher plant species used in its preparation.** Modeled distribution of the two most widely prepared species of pitcher plants, *Nepenthes ampullaria* and *Nepenthes mirabilis,* based on verified herbarium specimen localities. Beige shading indicates co-occurrence of species. Yellow triangles indicate visited marketplaces. Circles denote areas where the consumption or sale of the glutinous rice snack was identified through social media.

In October and November 2014, we worked throughout the two states of Malaysian Borneo, Sabah and Sarawak, with three different questionnaires made for those that had eaten the snack, those that were vendors selling the snack on a market and those who offered more advanced knowledge of the preparation and tradition. We started our research at nine outdoor markets (Figure 1), where we surveyed market customers and vendors by first asking if they recognized a laminated photograph of the sticky rice dish cooked in *Nepenthes,* and then utilized snowball sampling [18] to identify additional areas, markets or informants. On the markets where no pitcher plant snacks were sold, we presented the photograph of the snack to a target of half of the vendors and customers (total n = 299) with the aim of encompassing diversity in tribe, gender and age. Upon recognition, informants were asked to answer our short ‘Market’ questionnaire designed for customers and vendors who were not selling the snack themselves (n = 11). This included questions about the frequency of their consumption of the snack, the reason they thought rice was cooked inside a *Nepenthes* pitcher, information about the ingredients used to make the dish and the species of *Nepenthes* used in cooking. To identify the species of *Nepenthes* used to make the snack, we showed a laminated photo series of the five regionally growing species to all informants, which offered a portable and convenient *ex situ* method of plant identification [18] with high rates of consistent plant recognition [19]. All vendors found selling the sticky rice snack were asked to complete a ‘Vendor’ focused questionnaire, which additionally asked about the harvesting of the pitchers and market sales.

Further, we prepared the snack along with an indigenous Bidayuh family from Sarawak, Malaysian Borneo by participant observation of the cooking process. With this, we also conducted a more detailed ‘Expert’ ethnobotanical questionnaire with more depth in the history, culture and preparation of the glutinous rice snack cooked in *Nepenthes* pitchers. The cook-along occurred in the informant’s homes in Bau in English. Two informants met whilst conducting our field study in Malaysia and ‘friended’ via Facebook also completed ‘Expert’ questionnaires. One questionnaire was mailed and returned via Facebook in English. The second questionnaire was conducted in Malay in Kuching by a local interpreter who then translated the information to English.

This study complies with the International Society of Ethnobiology (ISE) Code of Ethics, and all participating market vendors and customers were informed of the objectives of our research and questionnaires prior to beginning the interviews. Only those that agreed to participate were interviewed.

### Predicting distribution of species uncovered through social media

We modeled the potential distribution of the two prominent species used in preparation that were uncovered in our social media investigation, *Nepenthes ampullaria* Jack and *Nepenthes mirabilis* (Lour.) Druce. These areas of predicted distribution are based on the realized ecological niche preferences of known localities.

To build the ecological niche models (ENMs), we combined localities with soil data derived from the International Soil Reference and Information Centre (ISRIC) [20] and climate data from WorldClim (http://www.worldclim.org/) at a spatial resolution of 5 arc-minute Environmental Systems Research Institute (ESRI)-grids. We selected a set of soil and climate variables that strongly indicated distribution by using presence localities of all botanical collections from Southeast Asia within our data records. Selection of 8 bioclimate and 10 soil variables was based on the ecology of *Nepenthes* and highest vector loading using Principal Component Analysis (PCA) in R. Variables correlated (|Spearman rho| > 0.7) to these highest predicting variables were excluded. In the R environment [21], we used MaxEnt version 3.3.3 k [22,23] to model the projections of the potential distributions. MaxEnt uses presence-only data to predict approximate species distribution based on a probability distribution of maximum entropy, an approach that has been shown to outperform other modeling algorithms [23,24]. We trained the models using a background sample of all plant material collections from our data sources within Southeast Asia (92°E-165°E, 15°S-22°N), and projected the distributions onto phytogeographical subareas [25]. Geographic delimitation minimizes over-estimating distribution to islands with suitable abiotic conditions but no recorded species presence, as the sea is a likely dispersal barrier [26,27]. We present the two species using the 10% training presence threshold in MaxEnt for visual ease. This feature presents a binary visualization of areas encompassing 90% of the predicted distribution area.

### Pitcher fluid measurements

In order to assess the possibility of a chemical contribution of the pitcher to the stickiness of the glutinous rice, we measured pH and presence of amylase, an enzyme that catalyzes the hydrolysis of starch into sugars, which is known to break down the starch present in glutinous rice [28]. Amylase activity was measured in fluid of well-developed but still unopened pitchers in order to control for variation due to rainwater dilution or insect capture contamination. The pH measurements were made with CholorpHast® pH-indicator strips, and amylase starch hydrolysis was tested with iodine. *Nepenthes* pitchers have glands that secrete digestive fluid, which breaks down their captured prey [29]. Through the in depth questionnaires, we found that pitcher fluid was not used in the cooking process, however, there was still potential that glands release substance while they were being steamed during preparation. We therefore measured the pH of distilled water steamed in three *N. ampullaria* pitchers in ten-minute increments for the standard one-hour steam identified in our cook-along. To ascertain the pH needed to alter rice viscosity, we cooked white rice in acidic acid-amended cooking liquid with a pH of 7, 6, 5 or 4. We blended the rice after cooking and measured viscosity with a Brookfield High Shear cap2000+ [30].

## Results and discussion

Throughout Malaysian Borneo, over 300 people were approached about the glutinous rice snack made with *Nepenthes* pitchers, making this the most extensive ethnobotanical study of pitcher plants conducted so far. *Nepenthes* were identified throughout Malaysian Borneo as *peruik kera,* and the snack as *nasi pulut* (white rice) in *peruik kera.* Two alternative names for the snack were given by two Bidayuh informants; *klimuoh* from one informant on the Serian Central market and *tramuo* from a family in Bau.

### Consumers of carnivorous plants are of indigenous tribal decent

In our investigations, all participants that recognized this snack via our photographs were of indigenous tribal decent save for one Chinese immigrant who had purchased the snack on a Bidayuh market, suggesting that this preparation of *Nepenthes* pitchers has roots in the indigenous culture of plant use. We showed our identification card to over 300 customers and vendors on nine market locations throughout Malaysian Borneo (Figure 1), and the snack was recognized by either vendors or customers on all but two markets. On the markets where the snack was not sold, participants who had eaten the snack identified themselves as belonging to the Bidayuh, (n = 2) Dyak, (n = 4) or Kadazandusun (n = 4) tribes. Those of Iban and Orang Ulu tribes did not identify with the photographs of the snack in our survey. In the two markets with vendors selling the snack, pitchers were being sold and prepared by Bidayuh families (n = 11). Bidayuh (n = 3) and Kadazandusun (n = 1) tribal families that were not vendors on the market identified themselves as making the snack in their homes. There was also a Javanese/Malay family (n = 1) that was taught how to make the snack from Bidayuh friends. The three Bidayuh families answered our most detailed questionnaire. Both the Bidayuh and Kadazandusun tribes are non-nomadic lowland tribes of Sarawak and Sabah, respectively [16]. The Bidayuh, previously identified as Land Dyak, and the Kadazandusun are both egalitarian societies, which distinguishes them from the more highland tribes of both states. Tribes not positively identifying the sticky rice snack also lack documentation of such use in social media or descriptive ethnobotanical analyses [19], suggesting that it is at the least not as widely or currently used in their present day culture. Save for the Kelabit tribe of the Northern Bario Highlands, all tribes identified as having ties with the snack through social media were approached in our market surveys. We acknowledge that some people may not have shared knowledge with us due to language barrier or fear, as it was expressed to us in the markets of Sabah that these plants are protected and can not be legally harvested for such use. No such fear was voiced in any markets of Sarawak, which in general had more open communication. Indeed the United Nations Declaration of the Rights of Indigenous Peoples [30] and the more regional Sarawak Law and Forest Ordinance of 1958 [31] protects the preservation of the cultural heritage in using CITES protected material, such as *Nepenthes,* as long as it is not traded internationally without permits.

The preparation of carnivorous plants also reaches into other traditional cultures. Norwegians douse *Pinguicula vulgaris* L.*,* appropriately common-named ‘butterwort’, leaves in cow’s milk to make a thick sour milk called *tjukkmjølk* [32]. Southwestern Australian aborigines consumed *Drosera* spp. roots [33], and Europeans distilled large quantities of *Drosera* spp. in a drink called *Rosa Solis*, which was promoted to ‘stir up lust’ in those who drank it [34]. Even the aquatic *Utricularia vulgaris* L. has edible turions and leaves [35].

### Species used are common and sustainably harvested

The species used for glutinous rice preparation revealed in social media coincided with those found in traditional ethnobotanical analysis. *Nepenthes ampullaria* and *N. mirabilis* were the two species most identified through social media and are widely distributed (Figure 1). For these two species, our ecological niche models predicted potential distribution with significant confidence (*p* < 0.05). Amazingly, the distribution predictions combined with the information uncovered through social media reveals that pitchers of *N. mirabilis* are only prepared in areas where *N. ampullaria* is not distributed (Figure 1), indicating that *N. ampullaria* is the preferred species for preparation. Participant interviews identified *N. ampullaria* (n = 24), *N. bicalcarata* (n = 2), *N. gracilis* (n = 2), *N. mirabilis* (n =1) or *N. veitchii* (n = 1) as species used for preparation, and *N. ampullaria* was the only species prepared for sale on the market. All market and cook-along preparations filled only *N. ampullaria* pitchers and selectively chose pitchers of this species that had developed sturdy trap walls, not yet too brittle from age. Interviews indicate that the identified species of use were the same as those prepared by the mothers and grandmothers that taught them how to cook the dish.

The most prevalently used *Nepenthes* species are located within lowland areas, where they are easily accessible to tribal villages and harvested in such a way that this does not threaten the persistence of local populations, i.e. by cutting off selected pitchers only and leaving the remainder of the plant intact so that it is sustained by the remaining and newly produced pitchers. Wild collection in this way ensures the future of the food-source for the people for which it provides. This system of sustainable harvesting is a mainstay of indigenous natural resource consumption as indigenous tribes have long relied on wild collections from their environment [36].

### Preparation of the dish changed when modern kitchen utensils became available

**Figure 2:**
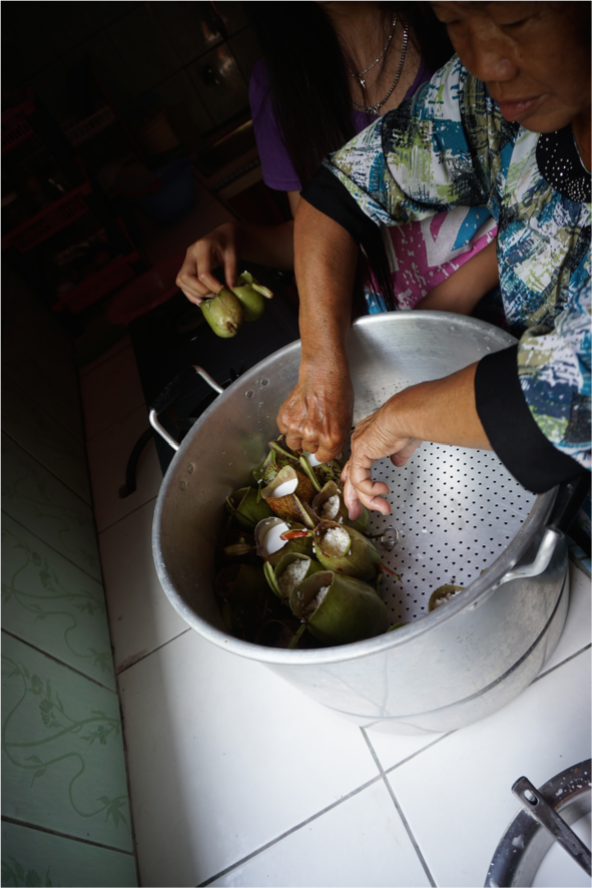
**Modern preparation of glutinous rice snack prepared in** ***Nepenthes ampullaria*** **pitchers.** Pitchers placed in steamer by indigenous Bidayuh family of Bau, Malaysia.

**Figure 3:**
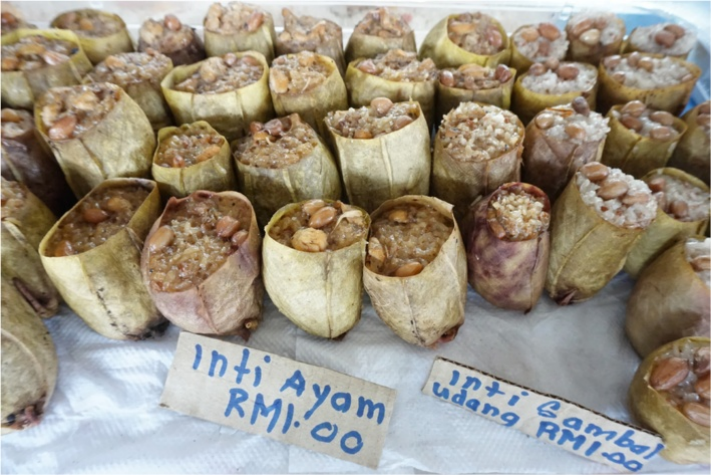
**Glutinous rice snack prepared in** ***Nepenthes ampullaria*** **pitchers for sale in Sarawak, Malaysia.** This photo shows two variations of the snack made with chicken (left) and prawn chili paste (sambal) (right) with peanuts on top. Each pitcher sells for one Malaysian Ringgit on this Kampung Duyoh roadside market.

Another remarkable interview suggests a more historical preparation of the sticky rice snack. One informant took a highland trek with tribal guides who harvested *Nepenthes* pitchers, coated them in a thick mud and then placed them directly on the coals of the fire to cook rice. The sterility of unopened pitchers [37] might have initially made *Nepenthes* an attractive vessel option for serving food in times when kitchen hygiene was more cumbersome. Modernized cooking methods and supplies allow for deviations to this more rudimentary approach, as all of our ‘Experts’ steamed the rice snack in large batches, sometimes upwards of hundreds of pitchers at a time, in aluminum pots over an electric cooktop (Figure 2). The basic recipe consists of glutinous hill rice or Thai white glutinous rice cooked in coconut milk. Additions of *sambal udang* (prawns cooked in chili peppers), *rousong* (a dried meat product), chicken, peanut or pandan leaf were found on the Bau Wet market and the Kampung Duyoh Roadside market (Figure 3).

### Chemical attributes of the pitchers do not seem to influence rice preparation

Glutinous rice has inherent properties that make it sticky. The dominant starch, amylopectin, has a low gelatinization temperature, giving the moist, sticky texture so highly sought after in many dishes across Asia [38]. Although some food preparations, like the addition of vinegar to sushi rice in Japan [39], contribute to rice texture and stickiness, we found no indication that the *Nepenthes* pitcher might be adding to the stickiness of the rice. No amylase activity was detected in our test, the pH remained unchanged throughout the cooking process and the pH needed to actually increase viscosity was measured to be a pH of at least 3, which was a far deviation of the pH of 7 recorded in our cooking experiment.

### Pitchers are convenient, biodegradable rice containers

Throughout Southeast Asia, packaging food in natural materials was the default before the introduction of waxed papers, plastics and aluminum containers. Plants used as food containers like the world-spread calabash (*Lagenaria siceraria* (Molina) Standl.) [40,41] and cornhusks (*Zea mays* L.), which wrap Mexican tamales, are especially well known as they are still commonly used. For glutinous rice, bamboo (*Bambusa* spp*.*) and banana leaf (*Musa* spp.) are the plants most frequently used as packaging, often with elaborate and beautiful design [14]. In Thailand and Laos it is common to cook sticky rice in green bamboo directly on an open fire. The charred bamboo package is served and then peeled off to eat the glutinous rice inside [38].

**Figure 4:**
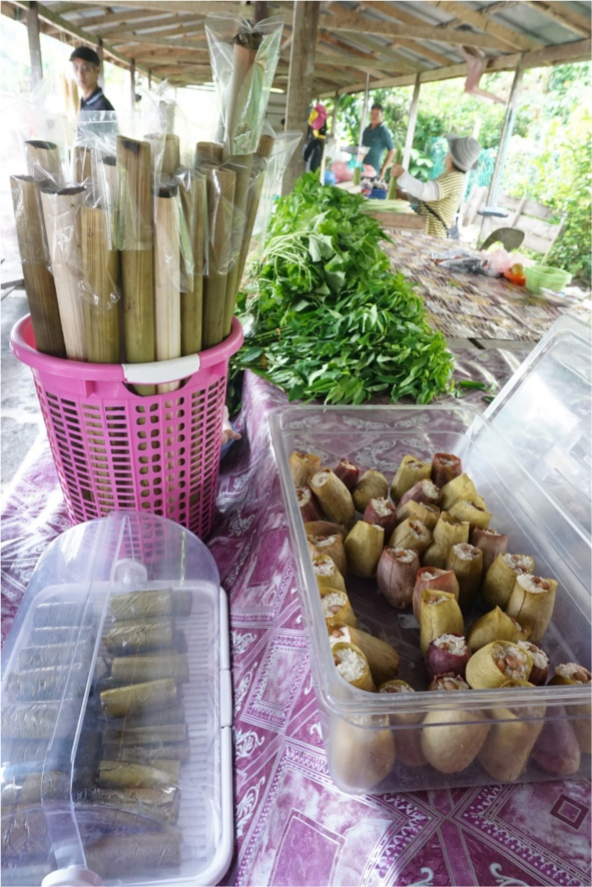
**Presentation of glutinous rice snacks prepared inside traditional packaging in Sarawak, Malaysia.** At the Kampung Duyoh roadsite market, sticky rice was prepared inside *Nepenthes ampullaria* pitchers (right) and within the leaf and culm of bamboo (*Bambusa* spp.) (left).

Like these, *Nepenthes* pitchers offer a charming folk packaging that protects the food and has an appeal unmatched by synthetic vessels (Figure 4). In line with their attractive presentation, participants and social media identified that the glutinous rice snack filled inside pitchers were often served to celebrate Ramadan, election parties and harvest festivals. When limitation of serving vessels might otherwise prohibit larger gatherings, pitchers offer an option to feed many people at one time. *Nepenthes* pitchers are convenient, biodegradable containers. In an age in desperate need of waste reduction and resource conservation [42], containers like these are a great solution to a growing environmental global problem. Edible packaging trends as one of the top innovations that will change our lives [43], and the buzz around Wikipearl™ shows that consumers desire options that are more eco- and health-friendly.

### Added value of social media for carrying out interview-based ethnobotany research

Social media provided a valuable tool in our research, allowing us a present day glimpse of the accessibility of the *Nepenthes* snack and assisting in our post-fieldwork data collection. ^©^Flickr, ^©^Pinterest and ^©^YouTube unearthed present day localities of where we might find the snack and revealed a modern excitement about this heritage-rich food. The revival of food culture through social media pulses around the globe, where blogs, Tweets and Instagrams of your favorite food, recipe and restaurant have created a virtual food community accessible by all linked to technology [44]. The cronut craze of 2013 brought so much attention via social media that fans flocked to its croissant/donut New York birth-site and even camped outside its doors in hopes of a deep-fried reward. Because of this, we suggest that social media might be used as a promotional weapon for heritage-rich snacks like the *Nepenthes* glutinous rice dish. One ^©^YouTube video, which originally aired as a popular Malaysian children’s cartoon highlighting the traditional *Nepenthes* snack, already has over 4.2 million views (http://youtu.be/9Y2ZisNsOjE). The presence of this dish in social media is a modern version of story telling and passing on of traditional knowledge.

### Benefits of a modern culture keeping traditional ways

Carrying on the tradition of food promotes cultural, personal and even biodiversity health. This interplay between nature and culture has become even more apparent as we feverishly attempt to hold on to both before they are lost [45]. Recognizing the ‘fast disappearing’ traditional knowledge of indigenous communities, the Sarawak Biodiversity Council in Malaysian Borneo focuses their efforts on preserving their valuable heritage. Malaysian Borneo boosts a quickly growing economy and global vibrancy [46]. There is a culture of eating out in modern society, with MacDonald’s restaurants, KFCs and Burger Kings lining high traffic streets and shopping malls. Echoing this is a 300% increase in the number of obese persons in Malaysia, up from just 4.4% in 1996 to 15% in 2011 [47]. If Malaysia grips tightly onto their food heritage, they might save themselves from the health consequences indebted now in the developed western world. A study with the Pima Indians of Mexico indeed showed that populations with diets more reflective of their traditional ways had lower incidence of obesity and non-insulin dependent diabetes [48].

In addition to the benefits of human health, preservation of traditional food enhances the link that people have with their natural environment and sustainable forest practices [49]. This need harkens radically in Borneo, where an average of 5% of the total forested area is lost to deforestation each year [50]. Maintaining the connection between nature and local people strengthens the value placed on local forests and increases the potential that communities will cry for preservation of these sites despite drives for more immediate, but temporary economic growth [45].

## Conclusion

The human drive to protect heritage resounds in the numerous museums throughout the world, in the detailed stories of histories and through the food served at our tables. Market surveys and social media resources revealed that the glutinous rice snack is still produced and consumed today among traditional communities throughout Southeast Asia. Small-scale commercial sale of glutinous rice snacks in local markets in Sarawak is less widespread, but vendors report easy access to pitchers and consistent sales of their cooked product. The tradition of cooking glutinous rice in *Nepenthes* pitcher plants in Malaysia likely carries from a time when cooking vessels were more limited and persists in tribal culture today because of value placed on maintaining cultural heritage, appeal and the inherent intrigue of these plants. Social media proved a valuable tool in our research, and we endorse its further use in ethnobotanical investigations and spread of knowledge. The documentation and gathered data weigh important for the ethnobotanical preservation of Bornean tribal culture and traditions.

## References

[1] Popkin BM (2006). Global nutrition dynamics: the world is shifting rapidly toward a diet linked with noncommunicable diseases. Am J Clin Nutr.

[2] Davey TM, Allotey P, Reidpath DD (2013). Is obesity an ineluctable consequence of development? A case study of Malaysia. Public Health.

[3] Popkin BM, Adair LS, Ng SW (2012). Global nutrition transition and the pandemic of obesity in developing countries. Nutr Rev.

[4] Pilgrim SE, Cullen LC, Smith DJ, Pretty J (2008). Ecological knowledge is lost in wealthier communities and countries. Environ Sci Technol.

[5] Jaiswal V (2010). Culture and ethnobotany of Jaintia tribal community of Meghalaya, Northeast India - A mini review. Indian J Tradit Knowl.

[6] Mey FS (2010). Cambodian of Natural History. Cambodian J Nat Hist.

[7] Perry LM, Metzger J (1980). Medicinal plants of East and Southeast Asia: attributed properties and uses.

[8] Balangcod TD, Balangcod AKD (2011). Ethnomedical knowledge of plants and healthcare practices among the Kalanguya tribe in Tinoc, Ifugao, Luzon, Philippines. Indian J Tradit Knowl.

[9] Wiart C (2006). Medicinal plants of the Asia-pacific: drugs for the future.

[10] Likhitwitayawuid K, Kaewamatawong R, Ruangrungsi N, Krungkrai J (1998). Antimalarial naphthoquinones from *Nepenthes thorelii*. Planta Med.

[11] Sumsakul W, Plengsuriyakarn T, Chaijaroenkul W, Viyanant V, Karbwang J, Na-Bangchang K (2014). Antimalarial activity of plumbagin *in vitro* and in animal models. BMC Complement Altern Med.

[12] Milliken W (1992). Ethnobotany of the Yali of West Papua.

[13] Christensen H (2002). Ethnobotany of the Iban & the Kelabit.

[14] Laistrooglai A, Mosikarat P, Wigran M (2000). Packaging design with natural materials: a study for conservation.

[15] Pietropaolo J, Pietropaolo P (1986). Carnivorous plants of the world.

[16] Deavin G, Denis JI, Djandam AM, Dols H, Lajumin P, Lanting AY, Lasimbang R, Spence J, Widjojo N: The Human Heart of Borneo. WWF Heart of Borneo Global Initiative; 2012

[17] Clarke C, Wong KM (1997). Nepenthes of Borneo.

[18] Martin GJ (1995). Ethnobotany: a methods manual.

[19] Thomas E, Vandebroek I, van Damme P (2007). What works in the field? a comparision of different interviewing methods in ethnobotany with special reference to use of photographs. Econ Bot.

[20] Batjes NH: ISRIC-WISE derived soil properties on a 5 by 5 arc-minutes global grid (ver. 1.2). 2012.

[21] Matloff N: R for Programmers. 2008:1–104.

[22] Phillips SJ, Anderson RP, Schapire RE (2006). Maximum entropy modeling of species geographic distributions. Ecol Modell.

[23] Elith J, Phillips SJ, Hastie T, Dudík M, Chee YE, Yates CJ (2011). A statistical explanation of MaxEnt for ecologists. Divers Distrib.

[24] Aguirre-Gutiérrez J, Carvalheiro LG, Polce C, van Loon EE, Raes N, Reemer M (2013). Fit-for-purpose: species distribution model performance depends on evaluation criteria – dutch hoverflies as a case study. PLoS One.

[25] Van Welzen PC, Parnell JA, Slik JF (2011). Wallace’s Line and plant distributions: two or three phytogeographical areas and where to group Java?. Biol J Linn Soc.

[26] Schupp E, Milleron T, Russo S (2002). Dissemination limitation and the origin and maintenance of species- rich tropical forests. In seed dispersal and frugivory: ecology, evolution and conservation.

[27] Holt R, Barfield M, Gomulkiewicz R, Sax D, Stachowicz J, Gaines S (2005). Theories of niche conservatism and evolution: could exotic species be potential tests?. Species invasions insights into ecol evol biogeogr.

[28] Limpisut P, Jindal VK (2002). Comparison of rice flour pasting properties using brabender viscoamylograph and rapid visco analyser for evaluating cooked rice texture. Starch-Starke.

[29] Tökés ZA, Woon WC, Chambers SM (1974). Digestive enzymes secreted by the carnivorous plant *Nepenthes macferlanei* L. Planta.

[30] United Nations. United Nations Declaration on the Rights of Indigenous Peoples. 2008(March)

[31] Human Resource Management Unit, Chief Minister Department of Sarawak in cooperation with State Attorney General. Laws of Sarawak: Forest Ordinace. Kuching; 1958.

[32] Nilsson R, Nilsson G (1958). Studies concerning Swedish ropy milk: The antibiotic qualities of ropy milk. Arch Mikrobiol.

[33] Hammond J (1933). Winjan’s People.

[34] Plat H: Delightes for Ladies: To Adorn Their Persons, Tables, Closets, and Distillatories with Beauties, Banquets, Perfumes and Waters. London: Hymfrey Lownes; 1609

[35] Chiej R (1984). Encyclopedia of medicinal plants.

[36] Bharucha Z, Pretty J (2010). The roles and values of wild foods in agricultural systems. Philos Trans R Soc London.

[37] Buch F, Rott M, Rottloff S, Paetz C, Hilke I, Raessler M (2013). Secreted pitfall-trap fluid of carnivorous *Nepenthes* plants is unsuitable for microbial growth. Ann Bot.

[38] Schiller J, Hasadong, Rao S, Inthapanya P, Schiller J, Chanphengxay M, Linquist B, Rao S (2006). Glutinous rice in Laos. Rice Laos.

[39] Odahara M, Sokooshi H, Takahashi T, Okadome H, Ohtsubo K (2004). The effect of sushi vinegar on texture of sushi rice before and after storage under low temperature. Nippon Shokuhin Kagaku Kogaku Kaishi.

[40] Price S: When is a calabash not a calabash? In New West Indian Guid/Nieuwe West-Indische Gids. Volume 56. Leiden; 1982:69–82.

[41] United States Environmental Agency (2013). Reducing wasted food & packaging, a guide for food services and packaging.

[42] Buzz WikiPearl^TM^. 2014. RSS. N.p., n.d. http://wikipearl.com/buzz/. Accessed 06 Jan 2015.

[43] Rousseau S (2013). Food and social media: You Are What You Tweet.

[44] Pilgrim S, Pretty J (2010). Nature and culture: rebuilding lost connections: Sarah Pilgrim, Jules N. Pretty: 9781844078219: Amazon.com: Books.

[45] United Nations. World Population Prospects: 2000 Revision Population Database. http://esa.un.org/unpd/wpp/index.htm. Accessed Jan 10 2015.

[46] Institute for Public Health Ministry of Malaysia. National Health and Morbidity Survey 2011. 2011.

[47] Ravussin E, Valencia ME, Esparza J, Bennett PH, Schulz LO (1994). Effects of a traditional lifestyle on obesity in Pima Indians. Diabetes Care.

[48] Alexiades MN, Peters CM, Laird SA, Binnqüist CL, Castillo PN (2013). The missing skill set in community management of tropical forests. Conserv Biol.

[49] Miettinen J, Shi C, Liew SC (2011). Deforestation rates in insular Southeast Asia between 2000 and 2010. Glob Chang Biol.

[50] Pretty J, Adams B, Berkes F, de Athayde SF, Dudley N, Hunn E (2009). The intersections of biological diversity and cultural diversity: towards integration. Conserv Soc.

